# Preclinical Rheumatoid Arthritis: Pathogenesis, Risk Stratification, and Therapeutic Interception

**DOI:** 10.3390/jcm15093283

**Published:** 2026-04-25

**Authors:** Yukina Mizuno Yokoyama, Ryu Watanabe, Mayu Shiomi, Ryuhei Ishihara, Yuya Fujita, Masao Katsushima, Kazuo Fukumoto, Yoichiro Haji, Shinsuke Yamada, Motomu Hashimoto

**Affiliations:** 1 Department of Clinical Immunology, Osaka Metropolitan University Graduate School of Medicine, 1-4-3, Asahi-machi, Abeno-Ku, Osaka 545-8585, Japan; 2 Department of Rheumatology, Daido Hospital, Nagoya 457-8511, Japan

**Keywords:** clinically suspect arthralgia, mucosal immunity, fibroblast, preclinical rheumatoid arthritis, prevention

## Abstract

Rheumatoid arthritis (RA) has traditionally been managed after the onset of clinically apparent synovitis; however, accumulating evidence indicates that disease-related immune abnormalities precede clinical diagnosis by several years. This preclinical phase is characterized by systemic autoimmunity, early musculoskeletal symptoms, and subclinical inflammation in genetically and environmentally susceptible individuals. In this review, we summarize current concepts regarding the pathogenesis, risk stratification, and therapeutic interception of preclinical RA. Particular attention is given to the mucosal origin hypothesis and to the roles of immunosenescence, peripheral helper T cells, and fibroblast-like synoviocytes in early disease evolution. Recent advances in clinical, serological, and imaging-based risk stratification have improved the identification of individuals at high risk of progression to clinical RA, and emerging intervention trials have shown that selected therapies may delay disease onset or reduce early inflammatory burden. Although complete prevention of RA has not yet been achieved, these findings support a paradigm shift from the treatment of established RA toward earlier, risk-adapted intervention before irreversible joint damage occurs. Future efforts should focus on refining predictive biomarkers, optimizing the timing and intensity of intervention, and establishing safe, individualized preventive strategies.

## 1. Introduction

The therapeutic landscape of rheumatoid arthritis (RA) has changed dramatically over the past two decades. With the widespread implementation of treat-to-target strategies using methotrexate (MTX), biologic DMARDs (bDMARDs), and targeted synthetic DMARDs (tsDMARDs), including Janus kinase (JAK) inhibitors, clinical remission has become an achievable treatment target for many patients [1,2]. Nevertheless, important unmet medical needs remain [3]. Approximately 10–20% of patients develop difficult-to-treat RA (D2T RA), a condition defined by persistent signs or symptoms suggestive of active disease despite treatment with at least two b/tsDMARDs [4,5,6]. In these patients, the clinical burden extends beyond progressive joint destruction to substantial deterioration in quality of life (QOL) and activities of daily living (ADL), underscoring the need for more effective strategies to alter the disease course at an earlier stage.

RA is now increasingly recognized as a continuum in which immunological abnormalities, driven by interactions between genetic susceptibility and environmental exposures, evolve over several years before synovitis becomes clinically apparent [7]. Within this framework, growing interest has focused on intervention during the phase preceding the development of clinical synovitis—that is, preclinical RA—with the aim of preventing or at least delaying the onset of overt disease [8]. This shift in perspective, from treating RA only after clinical onset to intercepting disease before its manifestation, represents a major paradigm change. However, true prevention has not yet been established [9].

This narrative review was based on a literature search of PubMed and related reference lists performed through March 2026, with emphasis on English-language studies relevant to preclinical rheumatoid arthritis, including pathogenesis, risk stratification, imaging, biomarkers, and interventional studies. Particular priority was given to recent cohort studies, clinical trials, and mechanistic investigations with translational relevance.

## 2. Definition and Staged Progression of Preclinical RA

Clinical RA is defined as the stage at which inflammatory arthritis is evident on physical examination and the patient meets established classification criteria, such as the 2010 American College of Rheumatology (ACR)/European Alliance of Associations for Rheumatology (EULAR) classification criteria [10]. By contrast, preclinical RA encompasses the period before the onset of clinically apparent synovitis, during which RA-related autoimmunity, symptoms, and/or imaging findings of subclinical inflammation may already be detectable [11]. Studies conducted since the 2000s have demonstrated that, in many individuals who later develop RA, anti-citrullinated protein antibodies (ACPAs), rheumatoid factor (RF), inflammatory cytokine abnormalities, and imaging evidence of synovitis or bone marrow edema can be identified several years or even more than a decade before clinical diagnosis [12,13,14,15,16]. Accordingly, the concept of preclinical RA has become established as an umbrella term for the phases preceding overt clinical disease.

In 2012, the EULAR Study Group proposed a five-stage framework for the development of RA [17]: (1) genetic risk factors for RA, characterized by the presence of genetic susceptibility, such as the *HLA-DRB1* shared epitope (SE), in the absence of symptoms or laboratory abnormalities; (2) environmental risk factors for RA, in which environmental exposures such as smoking increase disease risk, although RA-specific autoantibodies and inflammatory findings are not yet present; (3) systemic autoimmunity associated with RA, defined by the presence of autoantibodies such as ACPAs and RF and/or circulating biomarkers without musculoskeletal symptoms; (4) symptoms without clinical arthritis, in which symptoms such as arthralgia or morning stiffness are present despite the absence of clinically detectable synovitis; and (5) unclassified arthritis, in which inflammatory arthritis is clinically evident but the patient does not yet fulfill RA classification criteria.

**Figure 1:**
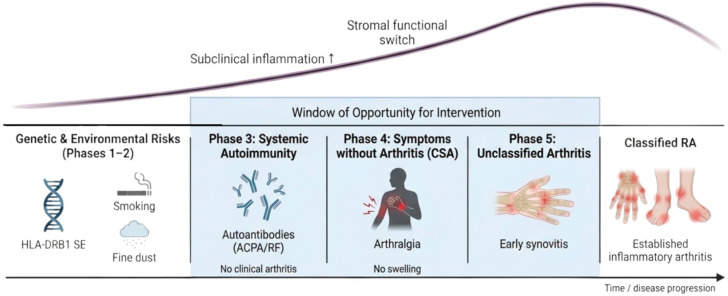
The multistep progression of preclinical rheumatoid arthritis and the window of opportunity for therapeutic intervention.

Within the fourth stage, the 2017 EULAR definition further identified a subgroup with symptoms suggestive of imminent progression to RA, termed arthralgia suspicious for progression to RA or clinically suspect arthralgia (CSA), and proposed this population as a key target for clinical studies and preventive intervention trials [18]. This stage-based perspective is indispensable for considering not only when to intervene, but also the appropriate intensity and invasiveness of intervention at each phase of disease evolution [19] (Figure 1).

Rheumatoid arthritis (RA) develops through a continuum initiated by interactions between genetic susceptibility, including the *HLA-DRB1* shared epitope (SE), and environmental factors such as smoking and fine particulate exposure. After the appearance of RA-related autoantibodies, including anti-citrullinated protein antibodies (ACPAs) and rheumatoid factor (RF), individuals may progress through clinically suspect arthralgia (CSA) and unclassified arthritis before developing clinically apparent RA. The stages spanning phase 3 (systemic autoimmunity associated with RA) to phase 5 (unclassified arthritis) are generally regarded as the principal window of opportunity for intervention before the development of clinically apparent RA.

## 3. Mucosal Origin Hypothesis in Preclinical RA

Mucosal surfaces constitute the interface between the external environment and the immune system and are considered major sites of early innate immune dysregulation in preclinical RA [20,21,22]. In particular, the intestinal, respiratory, and oral mucosae are continuously exposed to environmental factors and have therefore attracted considerable attention as potential sites where autoantibody production and epitope spreading may be initiated [23,24].

### 3.1. Gut Microbiota

In recent years, a growing number of studies have suggested that alterations in the gut microbiota, or dysbiosis, may contribute to the pathogenesis of preclinical RA. Among the taxa implicated, the genus Prevotella, and especially Prevotella copri, has received particular attention as a factor linked to genetic susceptibility and RA-related autoimmunity. Maeda et al. analyzed the gut microbiota of treatment-naive patients with RA and demonstrated that *P. copri* was significantly enriched compared with healthy controls. They further showed that colonization of SKG mice with *P. copri* exacerbated Th17 responses and arthritis, suggesting that *P. copri* may contribute to arthritis development through activation of the Th17 axis [25,26,27].

The relationship between host genetic background and the gut microbiota has also been examined in the TwinsUK and SCREEN-RA cohorts [28]. Individuals with a higher RA polygenic risk score showed a greater relative abundance of specific Prevotella amplicon sequence variants (ASVs), particularly Prevotella 7, and this tendency was more pronounced among carriers of the *HLA-DRB1* SE [28]. A significant correlation between Prevotella 7 and the SE was observed, suggesting that individuals with an SE-positive background may already harbor a Prevotella-dominant intestinal microbial profile during the preclinical phase of disease [28].

Moreover, even at the stage of ACPA-positive preclinical RA, differences in the gut microbiota may be associated with the risk of progression to clinically manifest RA [29]. Rooney et al. longitudinally followed anti-CCP-positive at-risk individuals and showed that those who progressed to RA had a significantly higher baseline abundance of fecal Prevotellaceae, particularly ASVs corresponding to *P. copri*, than those who did not progress [29]. In addition, individuals with higher levels of Prevotellaceae were more likely to develop RA during follow-up [29].

Taken together, these findings suggest that the gut microbiota may be involved primarily in the transition from preclinical RA to clinically apparent RA. Although no clinical trials have yet been established in this setting, microbiota-targeted therapeutic strategies, including probiotics, dietary interventions, traditional herbal medicines, and even fecal microbiota transplantation (FMT), are attracting increasing interest as potential preventive approaches [30,31,32].

### 3.2. Smoking and the Airway Mucosa

Smoking is one of the most well-established environmental risk factors for RA, particularly for ACPA-positive disease, and its effect is strongly modified by the presence of *HLA-DRB1* SE alleles [33,34]. The airway mucosa, especially the lung, has therefore been proposed as a key site in the early breach of immune tolerance. In smokers, lung tissue exhibits increased expression of peptidylarginine deiminase (PAD) 2 and PAD4, together with enhanced accumulation of citrullinated proteins, supporting the concept that smoking promotes a local microenvironment favorable to the generation of neoantigens [35]. These citrullinated proteins may subsequently trigger T-cell and B-cell responses, leading to the production of RA-related autoantibodies and, ultimately, the propagation of systemic synovial inflammation [21,36,37].

Recent evidence further strengthens the concept that the lung is not merely a site of environmental exposure but may serve as an immunologically active compartment linked to the joint. In a study of treatment-naive patients with new-onset ACPA-positive RA, Venken et al. demonstrated shared T-cell receptor clonotypes between bronchoalveolar lavage samples and inflamed synovial tissue, with this overlap being particularly prominent in smokers. Synovial samples from smokers, especially those carrying SE alleles, also showed greater enrichment of CD4-positive and CD8-positive T cells and increased expansion of T-cell clonotypes. These findings provide direct support for the hypothesis that smoking drives a lung-derived adaptive immune response that may subsequently traffic to the joint and contribute to early synovial inflammation [38].

An important question is at which stage of preclinical RA smoking exerts its greatest pathogenic effect. Studies in both asymptomatic individuals from the general population and individuals with CSA suggest that smoking is more closely associated with the earlier phases of disease evolution than with the final transition to clinically apparent inflammatory arthritis. In asymptomatic individuals, smoking is associated with ACPA positivity, whereas in the CSA phase, both smoking and the SE are linked to ACPA positivity. By contrast, progression from CSA to clinically apparent inflammatory arthritis appears to be associated primarily with the SE rather than smoking alone [39]. These observations suggest that smoking mainly contributes to the initiation and amplification of RA-related autoimmunity, whereas the transition from systemic autoimmunity to persistent synovitis likely requires additional determinants, including host genetic susceptibility and local joint-specific factors.

From a pathogenetic perspective, this stage-dependent pattern is informative. Smoking may not simply act as a nonspecific inflammatory trigger, but rather shape the earliest mucosal immune milieu in which loss of tolerance to citrullinated antigens occurs. Once autoimmunity has been established, however, subsequent progression to overt arthritis may depend on additional events such as epitope spreading, stromal cell activation, and the recruitment or retention of pathogenic immune clones within the synovium. In this context, the observation of a shared lung-joint T-cell repertoire provides a mechanistic framework linking cigarette smoke exposure, mucosal immune activation, and joint-specific inflammation.

This interpretation has clinical implications. Smoking cessation may be particularly relevant during the earliest phases of disease development, before clinically apparent arthritis emerges, and may be most effective when combined with other risk-stratification approaches, such as autoantibody profiling, genetic background, and imaging evidence of subclinical inflammation. Thus, the airway mucosa should be regarded not only as a site of environmental exposure, but also as a potential therapeutic and preventive target in preclinical RA.

### 3.3. Periodontal Pathogens

Periodontal pathogens, particularly *Porphyromonas gingivalis*, have also been implicated in the pathogenesis of preclinical RA because of their capacity to promote protein citrullination [40,41,42]. Hashimoto et al. prospectively followed patients with arthralgia suspected of RA who had not yet received antirheumatic therapy and investigated whether periodontitis and carriage of *Porphyromonas gingivalis* (Pg) were associated with subsequent RA development. They found that the frequencies of moderate-to-severe periodontitis and Pg positivity were significantly higher in patients who progressed to RA than in those who remained free of RA. These findings suggest that, in the preclinical phase, the presence of clinically manifest periodontitis may be more strongly associated with future RA diagnosis and the need for treatment intervention than Pg carriage alone, raising the possibility that control of periodontal inflammation may be important for RA prevention [43].

Mechanistic evidence further supports a contributory role of oral pathogens in RA-related autoimmunity. In an experimental rat model, oral exposure to *P. gingivalis* induced periodontitis together with systemic inflammation, bone erosions, and anti-CCP2-associated arthritis, providing in vivo evidence that periodontal infection may prime immune responses linked to inflammatory arthritis. These findings strengthen the concept that the oral mucosa may function not only as a site of chronic local inflammation but also as a source of immune priming capable of promoting RA-related autoimmunity [44].

In addition, Manoil et al. reported that higher total IgG titers against multiple periodontal pathogens were associated with ACPA positivity and concluded that exposure to a broader periodontal pathogen complex, rather than to a single bacterial species alone, may contribute to the development of ACPA-related autoimmunity [45]. This broader interpretation is important, because it suggests that periodontal dysbiosis may be more relevant than the presence of any single organism alone.

Recent longitudinal data also indicate that the clinical impact of periodontal disease may differ according to RA serological subtype. In individuals with CSA, tooth extraction used as a proxy for periodontal disease was associated with the development of ACPA-positive RA, but not ACPA-negative RA [46]. These findings suggest that periodontal inflammation may be more closely linked to pathways underlying ACPA-positive disease, consistent with the hypothesis that mucosal citrullination and loss of tolerance to citrullinated antigens are particularly relevant in this RA subset.

Overall, these observations indicate that periodontal disease should not be regarded simply as a binary exposure, but rather as a complex mucosal inflammatory state that may interact with host susceptibility to promote RA-related autoimmunity, particularly in ACPA-positive disease. From a clinical perspective, appropriate periodontal management may therefore represent one component of a broader preventive strategy in preclinical RA. More broadly, the findings reviewed in this section support a model in which environmental exposure and dysbiosis initiate RA-related autoimmunity at barrier sites. The subsequent transition to clinically apparent arthritis likely depends on amplification of systemic immune dysregulation and the emergence of tissue-localized stromal pathogenicity within the synovium.

## 4. Immune and Stromal Cells in Preclinical RA

### 4.1. Immunosenescence

At the preclinical stage of RA, immune abnormalities are already detectable in the peripheral blood [47], suggesting that systemic immune dysregulation begins before the onset of clinically apparent synovitis. Increasing evidence indicates that immunosenescence may contribute to this early phase of disease development rather than merely representing a consequence of chronic inflammation. Classical features of immune aging include thymic involution, reduced production of naive T cells, contraction of the T-cell receptor repertoire, telomeric attrition, and accumulation of late-differentiated effector T cells [48,49,50]. In RA, these changes appear to occur prematurely and may create an immune environment that is less capable of maintaining tolerance and more prone to autoreactivity [51].

From the earliest phase of disease, a process consistent with immunosenescence appears to be operative, characterized by reductions in naive T cells and recent thymic emigrants, together with elevated levels of proinflammatory cytokines such as interleukin-6 (IL-6) and tumor necrosis factor-α (TNF-α). As RA becomes established, this process is accompanied by accelerated accumulation of senescent T cells and age-associated B cells (ABCs). Recent stage-based studies further suggest that reduced thymic output and other immune-aging phenotypes can already be detected in drug-naive individuals with arthralgia or early RA [52], supporting the concept that immunosenescence may participate in disease initiation and facilitate the transition from preclinical autoimmunity to inflammatory arthritis.

Mechanistically, immunosenescence may promote RA-related autoimmunity through several pathways. Reduced thymic output may impair central tolerance by limiting the generation of a diverse naive T-cell pool, while contraction of the T-cell receptor repertoire may favor the survival and expansion of autoreactive clones. In parallel, the accumulation of senescent or late-differentiated effector T cells may enhance proinflammatory cytokine production and promote B-cell help, thereby facilitating autoantibody generation. In this framework, preclinical RA may be viewed as a state in which genetic susceptibility, environmental triggers, and age-related immune remodeling converge to break tolerance before joint inflammation becomes clinically evident.

IL-6 has also been identified as a key mediator promoting immunosenescence-like changes in fibroblast-like synoviocytes (FLS), and IL-6 inhibition has been shown to reduce the expression of senescence markers, including p16 and p21, while attenuating inflammation [53]. Collectively, these findings support the possibility that anticytokine strategies targeting immunosenescence-related pathways may represent a rational preventive approach in preclinical RA.

### 4.2. Peripheral Helper T Cells

Among the immune alterations observed in at-risk individuals, expansion of peripheral helper T (Tph) cells has emerged as a particularly relevant finding. According to the study by Inamo et al., increased Tph cells, which provide help for antibody production by B cells, represent a characteristic change shared across at-risk individuals with a high likelihood of developing RA, regardless of ACPA status. In addition, ACPA-positive high-risk individuals showed increased frequencies of CCR2^+^CD4^+^ T cells, which resemble Th17/Th22-like inflammatory cells, as well as PAX5^low^ B cells at a specific stage of differentiation. By contrast, in ACPA-negative high-risk individuals, expansion of CD15^+^ monocytes was observed in addition to the increase in Tph cells. These findings suggest that distinct but partially overlapping immune pathways may operate according to serological status, while activation of the adaptive and innate immune systems is already underway before the onset of clinically manifest disease [47].

Direct evidence demonstrating the presence of Tph cells in the synovium during preclinical RA remains limited. However, Tph cells are significantly increased in both synovial tissue and peripheral blood in early RA, and their behavior appears to differ from that of T follicular helper (Tfh) cells. In the synovium of early RA, Tph cells are found not only within lymphoid follicle-like structures but also more broadly at inflamed sites, where their localization in close proximity to germinal center B cells suggests that they may promote intra-articular autoantibody production from the earliest stages of disease [54].

Recent work has shown that Tph cells are composed of at least two functionally distinct subsets. Tph cells located within tertiary lymphoid structures express the transcription factor TCF1 and exhibit a stem-like phenotype with self-renewal capacity, while potently promoting B-cell differentiation and immunoglobulin production. By contrast, Tph cells outside tertiary lymphoid structures display a more effector-like phenotype and are preferentially associated with inflammatory niches containing proinflammatory macrophages and CD8-positive T cells [55]. These findings suggest that Tph cells may contribute both to local autoantibody production and to the persistence of synovial inflammation in early RA.

These findings have important implications for preclinical RA. Although comparable synovial data are still lacking at the preclinical stage, the expansion of circulating Tph cells in at-risk individuals, together with the central role of Tph cells in early synovial B-cell help, supports the hypothesis that this cell population may participate in the transition from systemic autoimmunity to tissue-localized inflammation. Therapeutic targeting of pathways highly expressed by Tph cells, such as programmed cell death protein 1 (PD-1) and inducible T-cell costimulator (ICOS), or blockade of Tph-derived interleukin-21 signaling, may therefore represent a strategy to suppress autoantibody production and local inflammation before the development of overt clinical arthritis [54,56,57].

### 4.3. Fibroblast-Like Synoviocytes

Fibroblast-like synoviocytes (FLS), the major stromal cell population in the synovium, are now recognized as key effectors of persistent inflammation and joint destruction in RA, acting not merely as structural cells but as functionally specialized pathogenic subsets that interact closely with immune cells and bone-resorbing pathways [58,59]. In this context, FLS have attracted increasing attention not only in established RA but also in the earliest phases of disease development.

Emerging evidence suggests that FLS abnormalities may already be present during the preclinical phase of RA. Synovial tissue and fibroblasts from individuals at this stage have been reported to exhibit persistent DNA damage and impaired DNA repair capacity compared with healthy controls, suggesting that genomic instability may contribute to the earliest steps of disease development. This fibroblast damage appears to be more pronounced in local microenvironments enriched with T cells, further supporting the notion that senescence-like FLS may participate in disease initiation and progression. Notably, the senolytic agent dasatinib has been shown to partially restore impaired repair capacity in these cells, raising the possibility that targeting senescent stromal cells before clinically overt inflammation becomes established may represent a preventive strategy [60].

As disease evolves, FLS appear to undergo a two-step functional switch that may be critical for chronicity [61]. The first step is an early loss of immunoregulatory capacity. FLS derived from healthy joints or from arthritis that subsequently resolves spontaneously are able to suppress lymphocyte adhesion to vascular endothelium. However, this suppressive function is already lost in FLS derived from very early RA, even within the first few months after disease onset. The second step is the acquisition of an actively pathogenic phenotype. FLS from established RA can directly activate endothelial cells and promote lymphocyte recruitment even in the absence of exogenous cytokine stimulation. In particular, FLS in very early RA appear to display a transitional phenotype that interferes with the natural resolution of inflammation, suggesting that this stage may represent a key turning point toward persistent disease.

In addition to these temporal changes, FLS also exhibit joint-specific characteristics [62]. FLS derived from different anatomical sites, such as the hands, hips, and knees, retain distinct transcriptional profiles and chromatin accessibility patterns both at baseline and under tumor necrosis factor stimulation, indicating the presence of a joint-imprinted FLS phenotype. In particular, FLS derived from hand joints show stronger responses to inflammatory cytokines and matrix metalloproteinases, suggesting a more aggressive phenotype. These findings may partly explain why hand joints are particularly susceptible to early and severe damage in RA.

**Figure 2:**
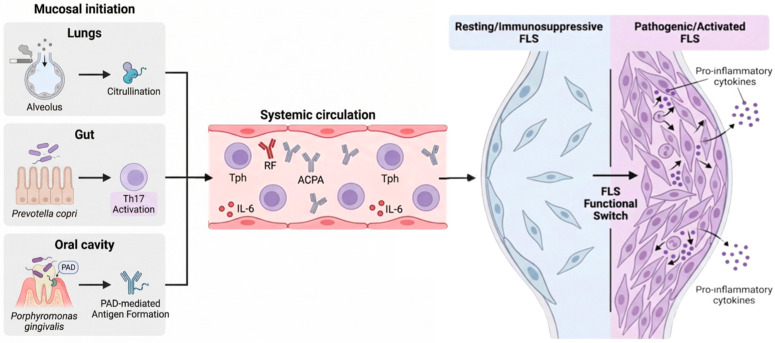
The mucosal origin hypothesis and functional switching of fibroblast-like synoviocytes in the pathogenesis of rheumatoid arthritis.

Taken together, these observations suggest that FLS are involved not only in the perpetuation of established synovitis but also in the earliest pathogenic events preceding clinically apparent RA. This concept has important therapeutic implications. In addition to established approaches such as interleukin-6 blockade and Janus kinase inhibition, early interventions directly targeting stromal cells—including cyclin-dependent kinase 4/6 inhibitors, fibroblast activation protein-targeted therapies, and senolytic agents—may emerge as key strategies in the prevention and treatment of preclinical RA [60,63,64] (Figure 2).

Autoimmunity in rheumatoid arthritis (RA) is thought to be initiated at mucosal surfaces, including the lung, gut, and oral cavity, through the interplay of environmental exposures and dysbiosis. In the lung, smoking promotes protein citrullination; in the gut, *Prevotella copri* has been implicated in activation of the T helper 17 axis; and in the oral cavity, *Porphyromonas gingivalis* contributes to the generation of autoantigens through its peptidylarginine deiminase (PAD) activity. These mucosal immune events are associated with systemic increases in peripheral helper T cells (Tph), rheumatoid factor (RF), anti-citrullinated protein antibodies (ACPA), and inflammatory cytokines such as interleukin-6. The transition from preclinical RA to clinical RA is characterized by a functional switch in fibroblast-like synoviocytes, from an immunoregulatory phenotype to a pathogenic phenotype marked by enhanced migratory capacity and inflammatory cytokine production.

## 5. Risk Stratification for Therapeutic Intervention

Not all autoantibody-positive individuals will ultimately develop RA; therefore, appropriate risk stratification is essential to avoid overdiagnosis and overtreatment. A multilayered assessment incorporating symptoms, serological markers, and imaging findings is required to identify those individuals who are truly at high risk, thereby enabling appropriate allocation of limited medical resources and minimizing unnecessary drug exposure.

### 5.1. EULAR-Defined Clinical Manifestations Suggestive of Progression to RA

In its 2017 report, EULAR not only proposed the definition of CSA but also identified this symptomatic pre-arthritis phase as the earliest clinically recognizable stage at which individuals at risk of RA may be detected. Using a data-driven and consensus-based approach, the taskforce defined seven clinical parameters that characterize arthralgia suspicious for progression to RA: symptom duration < 1 year, involvement of the metacarpophalangeal (MCP) joints, morning stiffness lasting ≥60 min, symptoms most severe in the early morning, a first-degree family history of RA, difficulty making a fist, and a positive squeeze test of the MCP joints. The combination of these variables showed good discriminative performance in the validation cohort, with an area under the receiver operating characteristic curve of 0.92. Importantly, the taskforce did not recommend a single cutoff, but instead showed that the presence of ≥3 parameters provided high sensitivity, whereas ≥4 parameters provided high specificity. Because these variables are readily assessable in routine clinical practice, they may serve as practical screening tools for identifying individuals with preclinical RA who warrant closer monitoring, particularly when combined with serological and imaging evaluation [18].

### 5.2. Serological Autoantibody Detection

ACPA and RF are the most powerful serological predictors of future RA, with ACPA generally showing greater specificity and higher likelihood ratios than RF. In particular, high-titer ACPA and double positivity for both ACPA and RF confer the greatest risk of progression [65,66]. The risk of developing RA increases in parallel with increasing ACPA titers, and when the ACPA level reaches three times the upper limit of normal, the probability of being diagnosed with RA within 3–5 years has been estimated at approximately 30–50% [7].

RF also has important predictive value, particularly when present at high titer. In a prospective cohort study from Denmark, healthy individuals with RF levels > 100 IU/mL had up to a 26-fold increased risk of developing RA, with a 10-year absolute risk as high as 32%. Even low-positive RF levels were associated with elevated risk, with hazard ratios of 3.6 for RF 25–50 IU/mL and 6.0 for RF 50.1–100 IU/mL compared with RF < 25 IU/mL [67]. These findings indicate that RF is not only a diagnostic marker, but may also identify individuals in the general population who are at substantially increased long-term risk of RA [68].

A recent systematic review and meta-analysis confirmed that RA-related autoantibodies substantially increase the risk of progression to RA, with ACPA being the strongest predictor overall. Anti-CCP2 remains the standard assay for identifying at-risk individuals, while anti-CCP3 may add prognostic value in selected populations. However, progression is not uniform among antibody-positive individuals: in patients with arthralgia, combined anti-CCP2 and IgM-RF positivity is associated with a cumulative RA incidence of approximately 35% at 12 months, whereas progression appears slower in asymptomatic relatives with a family history of RA [69].

A cohort study involving 1544 relatives of patients with RA applied a machine-learning model integrating genetic, serological, and clinical variables to predict RA onset. In that study, RF and arthralgia emerged as major predictive factors and showed high sensitivity, suggesting potential utility for identifying individuals at risk 6–18 months before disease onset and thereby facilitating earlier intervention [70].

More recently, a prospective cohort study of anti-CCP2-positive individuals with musculoskeletal symptoms showed that progression to ACPA-positive RA was associated not only with broader ACPA reactivity, but also with evidence of tissue inflammation and soluble immune activation markers, including ultrasound-detected tenosynovitis, elevated IL-6, and increased IL-15 receptor α [13]. These findings suggest that serological markers may be most informative when interpreted in combination with markers of tissue inflammation and immune activation, thereby allowing more precise identification of individuals at particularly high risk of progression.

### 5.3. Imaging

Even in the absence of clinically apparent joint swelling, inflammatory abnormalities detected by ultrasound or magnetic resonance imaging (MRI), such as synovitis, tenosynovitis, and bone marrow edema, are regarded as important predictors of progression. This condition, often referred to as subclinical inflammatory arthritis, has attracted particular attention in the context of preclinical RA. In ACPA-positive patients with arthralgia, the addition of imaging findings has been reported to increase the positive predictive value for progression to clinical arthritis to as high as 70–80% [65]. More broadly, MRI studies in CSA have shown that RA-related subclinical inflammation is not confined to synovium alone, but may involve multiple tissues, supporting the concept that imaging can capture a stage of tissue-specific inflammation before overt arthritis becomes clinically evident [71].

Among MRI abnormalities, tenosynovitis appears to be particularly informative. Detailed MRI studies have suggested that RA-specific inflammation in CSA is better identified by patterns that include tenosynovitis rather than synovitis alone, supporting the view that tendon sheaths may represent an important early target tissue in RA development [71]. In addition, tenosynovitis of the small joints has been shown to contribute to the symptom of morning stiffness, especially when present together with synovitis, linking subclinical imaging abnormalities to patient-reported symptoms [72].

At the same time, caution is warranted in interpreting imaging abnormalities in isolation. Longitudinal studies of CSA patients who did not progress to clinical arthritis showed that symptom resolution was accompanied by resolution of subclinical MRI inflammation in a subset of patients, indicating that at least some imaging abnormalities may be reversible. Conversely, persistent symptoms in non-progressors were not necessarily accompanied by persistently elevated MRI inflammation scores. These findings suggest that MRI abnormalities should not be interpreted as deterministic, but rather as dynamic features whose prognostic value depends on the broader clinical and serological context [73].

The anatomical extent of MRI assessment also deserves consideration. Although MRI-detected tenosynovitis in the feet has been associated with progression to RA, studies in undifferentiated arthritis have shown that adding foot MRI to hand MRI does not meaningfully improve predictive accuracy compared with hand MRI alone [74]. This suggests that, for pragmatic risk stratification, hand MRI may capture most of the clinically relevant prognostic information while reducing cost and complexity.

**Figure 3:**
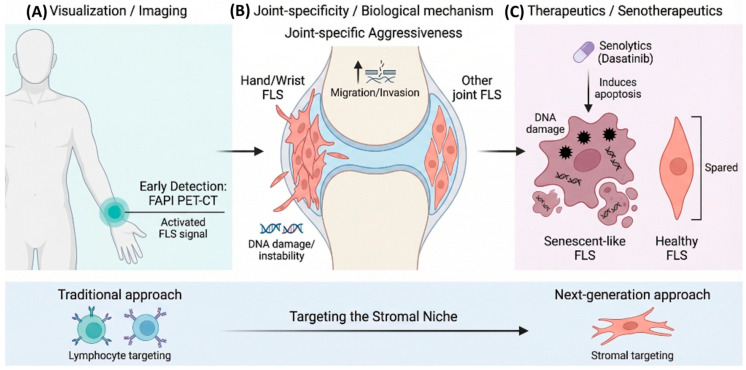
Next-generation diagnostic and therapeutic strategies targeting fibroblast-like synoviocytes. This figure illustrates emerging approaches that directly target stromal cells in rheumatoid arthritis. (**A**) Visualization: Fibroblast activation protein inhibitor (FAPI) positron emission tomography-computed tomography (PET-CT) enables noninvasive detection of activated fibroblast-like synoviocytes (FLS) before the appearance of clinically evident joint swelling and may therefore represent a novel imaging modality for the early diagnosis of preclinical rheumatoid arthritis. (**B**) Joint specificity: Fibroblast-like synoviocytes derived from specific joints, such as the hands, exhibit gene expression patterns associated with enhanced migratory and invasive properties compared with fibroblast-like synoviocytes from other anatomical sites, suggesting that joint-specific stromal programs may contribute to the selective distribution of joint involvement in rheumatoid arthritis. (**C**) Therapeutic targets: Senescence-like fibroblast-like synoviocytes induced by aging and chronic inflammation may amplify disease through the senescence-associated secretory phenotype. Senolytic agents such as dasatinib may selectively eliminate these pathogenic fibroblast-like synoviocytes and thereby inhibit disease progression through mechanisms distinct from those of conventional immunosuppressive therapies.

In addition, attempts have been made to visualize fibroblast-like synoviocyte activation in vivo during the preclinical phase of RA. In studies using the novel imaging modality fibroblast activation protein inhibitor (FAPI) positron emission tomography-computed tomography (PET-CT), increased FAPI uptake, presumed to reflect synovial fibroblast activation, was detected even in at-risk individuals without clinically apparent arthritis or joint swelling. Higher intra-articular signals were significantly associated with an increased future risk of developing RA, and uptake correlated closely with both ACPA levels and arthralgia, suggesting that imaging of activated fibroblasts may serve as a novel biomarker for predicting future disease onset [75] (Figure 3).

### 5.4. Pulmonary Abnormalities

Among individuals positive for RA-related autoantibodies, pulmonary abnormalities are frequently present even before the onset of clinically apparent arthritis. In one study, high-resolution computed tomography performed in subjects positive for anti-CCP antibodies and/or multiple RF isotypes, but without clinical arthritis, revealed airway abnormalities in approximately 70% of cases, including bronchial wall thickening, bronchiectasis, centrilobular opacities, and air trapping [76]. These pulmonary changes closely resemble those observed in early RA and are substantially more frequent than in healthy controls, supporting the hypothesis that RA-related autoimmunity may, at least in part, originate in the lung.

### 5.5. Gene Expression Profile

Gene expression profiling has also emerged as a potential tool for identifying individuals with preclinical RA who are at increased risk of progression to clinically apparent inflammatory arthritis. In a longitudinal cohort of patients with CSA, Niemantsverdriet et al. examined whole-blood RNA expression of 133 genes related to inflammation and immunity and found that several genes were differentially expressed between patients who did and did not develop inflammatory arthritis. Among these, lower expression of interleukin-7 receptor (IL-7R) and insulin-like growth factor 1 (IGF-1) was independently associated with progression, even after adjustment for established clinical predictors and autoantibody status [77]. Biologically, reduced IL-7R expression may reflect impaired T-cell homeostasis and diminished maintenance of a balanced naive and memory T-cell compartment, whereas lower IGF-1 expression may be linked to altered immune-metabolic regulation and tissue repair. Together, these changes are consistent with a state of early immune disequilibrium in which tolerance is weakened and progression to inflammatory arthritis becomes more likely.

These findings suggest that gene expression profiling may capture immunological changes that are not fully reflected by conventional serological markers alone. In particular, the association of IL-7R and IGF-1 with progression supports the notion that dysregulated T-cell homeostasis and immune activation are already present during the symptomatic pre-arthritis phase.

### 5.6. Risk-Adapted Intervention Framework

Taken together, these findings suggest that risk stratification in preclinical RA should not be viewed merely as a predictive exercise, but as the basis for a risk-adapted intervention strategy. Individuals with isolated symptoms or low-level serological abnormalities may be appropriate for surveillance and repeated assessment, whereas those with convergent high-risk features—such as CSA, high-titer ACPA/RF positivity, imaging-detected tenosynovitis or synovitis, or molecular evidence of immune activation—may represent the population most likely to benefit from preventive pharmacologic intervention (Table 1).

## 6. Therapeutic Interventions in Preclinical RA

The following agents are highlighted because they have been evaluated in prospective trials or well-defined at-risk cohorts, whereas evidence for other candidate interventions remains limited or insufficiently mature for meaningful comparison.

### 6.1. Methotrexate (MTX)

Methotrexate (MTX), the standard anchor drug for RA, exerts its therapeutic effects not only through its classical antifolate activity but also through broader anti-inflammatory mechanisms, particularly adenosine A2A receptor-mediated signaling [78]. In the preclinical phase of RA, MTX is expected to interfere with several key pathogenic processes, including: (1) the rise in inflammatory cytokines and acute-phase reactants; (2) expansion of the autoantibody repertoire driven by interactions between B cells and T cells; and (3) the recognition of citrullinated antigens by circulating ACPA, followed by immune-complex formation and complement- and Fcγ receptor-mediated local inflammation [79].

In the 2022 TREAT EARLIER trial, a 1-year course of MTX did not significantly prevent progression to clinical arthritis, but it resulted in sustained improvement in MRI-detected synovitis and tenosynovitis, as well as pain and physical functioning [80]. In a 4-year follow-up analysis published in 2024, MTX was suggested to reduce the risk of progression to RA in ACPA-negative individuals at increased risk of disease development [81]. Analyses of MTX responsiveness in patients with CSA have also shown that approximately 38% of patients experience a meaningful reduction in MRI-detected subclinical inflammation, accompanied by improvements in pain and physical function, with particularly favorable responses observed in those with multifocal tenosynovitis or osteitis, whereas patients with isolated synovitis or lower-risk profiles appear less likely to benefit [82]. These findings indicate that baseline MRI assessment is important not only for risk prediction, but also for predicting therapeutic response.

A 2025 cost-effectiveness analysis of the TREAT EARLIER trial further showed that a fixed 1-year course of MTX in arthralgia at risk for RA was associated with better work productivity, lower healthcare costs, and improved quality of life over 2 years, and was considered cost-effective from both healthcare and societal perspectives [83]. Taken together, MTX does not appear to completely prevent RA onset overall, but in selected high-risk individuals it may delay progression and ameliorate clinically relevant disease-related outcomes.

### 6.2. Abatacept (ABT)

Abatacept (ABT) is a fusion protein consisting of the extracellular domain of cytotoxic T-lymphocyte-associated antigen 4 (CTLA4) linked to the Fc portion of human immunoglobulin G1 (CTLA4-Ig). It binds with high affinity to CD80/CD86 on antigen-presenting cells and thereby competitively inhibits CD28-mediated costimulatory signaling [84,85]. Beyond this canonical mechanism, ABT has been reported to reduce circulating Tfh cells as well as Tph cells, findings that are likely relevant to the immunopathogenesis of preclinical RA [86]. Notably, this appears to contrast with tumor necrosis factor inhibitors, which can reduce circulating Tph cells but do not substantially decrease circulating Tfh cells [86], suggesting that costimulatory blockade may more effectively suppress germinal center-related immune activation.

In the 2024 APIPPRA trial, high-risk individuals with inflammatory arthralgia and either double positivity for ACPA and RF or high-titer ACPA positivity were treated with weekly subcutaneous ABT for 1 year. Abatacept significantly reduced progression to clinical RA not only during treatment but also at 24 months, and improved arthritis-free survival compared with placebo [87]. Similarly, in the ARIAA trial, patients with ACPA-positive arthralgia and MRI evidence of bone marrow edema, synovitis, or tenosynovitis in the hands showed sustained improvement in MRI scores and reduced risk of RA development after a 6-month course of ABT [88].

Longer-term follow-up from the ALTO study, which tracked APIPPRA participants for up to 8 years, showed that a 1-year course of ABT delayed progression to RA by as much as 4 years, with the most pronounced benefit observed in individuals with broader autoantibody positivity. No new safety signals were identified, and tolerability remained generally favorable [89]. However, because the difference in cumulative incidence gradually narrowed after treatment discontinuation, abatacept is currently best regarded as an intervention that delays disease onset rather than achieving complete prevention. Although its cost-effectiveness has not yet been fully established, abatacept remains one of the few interventions to have demonstrated a meaningful disease-interception effect in preclinical RA.

### 6.3. Rituximab (RTX)

Rituximab, an anti-CD20 monoclonal antibody, selectively depletes B cells through several mechanisms, including antibody-dependent cellular cytotoxicity (ADCC), complement-dependent cytotoxicity (CDC), and induction of apoptosis [90]. Given the central role of B cells in autoantibody production, rituximab has been investigated as a potential disease-interception strategy in preclinical RA.

In the 2019 PRAIRI trial, a single infusion of rituximab 1000 mg was compared with placebo in patients with arthralgia who were positive for both ACPA and RF. Although no significant difference was observed in the cumulative incidence of clinical arthritis over long-term follow-up, rituximab prolonged the time to arthritis onset by approximately 12 months, and the hazard ratio for progression at 12 months was reported to be approximately 0.45 [91]. A subsequent analysis published in 2024 further showed that rituximab did not produce significant improvements in quality of life, pain, or HAQ scores at 2 years, despite the observed delay in arthritis onset [92]. Thus, while rituximab may contribute to postponing disease onset, clear evidence of sustained prevention remains lacking at present.

### 6.4. Hydroxychloroquine (HCQ)

Hydroxychloroquine (HCQ) exerts its immunomodulatory effects primarily through inhibition of endosomal toll-like receptor signaling, a mechanism that has been well characterized in systemic autoimmune diseases such as systemic lupus erythematosus [93,94]. In established RA, HCQ has also been used as part of combination strategies; for example, the RACAT trial demonstrated that triple therapy with MTX, sulfasalazine, and HCQ was non-inferior to etanercept plus MTX in MTX-resistant RA [95]. In early RA, similar triple-therapy approaches have also been associated with outcomes comparable to biologic-based treatment in terms of quality of life, but at substantially lower cost [94].

However, results in the preclinical setting have been less encouraging. In the phase 2 STOP-RA trial, published in 2025, adults with elevated anti-CCP levels but no clinical arthritis received HCQ 200–400 mg/day for 12 months and were followed for 36 months. HCQ did not significantly reduce the incidence of clinical RA or delay its onset compared with placebo [96]. Although the safety profile of HCQ was generally acceptable, current evidence does not support its use as an effective primary preventive therapy in preclinical RA (Table 2).

The heterogeneous results of interventional trials may reflect differences in the stage and depth of immune network engagement at the time of treatment. Agents such as ABT, which target T-cell costimulation and thereby may influence upstream T-cell/B-cell interactions, could be more effective during the transition from systemic autoimmunity to clinically manifest arthritis. By contrast, therapies such as HCQ, which primarily modulate innate immune signaling, may be less effective once autoantibody maturation, epitope spreading, and tissue-localized inflammatory circuits are already established. This stage-dependent therapeutic responsiveness raises the possibility that preclinical RA contains a “point of no return,” beyond which disease interception becomes increasingly difficult.

### 6.5. Supplements and Lifestyle Interventions

Compared with pharmacologic interventions, supplements and lifestyle modification generally exert smaller effect sizes; however, they are attractive because of their favorable safety profile and broad applicability. In the VITAL trial, participants were randomized to vitamin D, omega-3 fatty acids, both agents, or neither, and were followed for 5 years. Vitamin D, with or without omega-3 fatty acids, significantly reduced the incidence of autoimmune diseases overall, and a reduction in RA incidence was also suggested, although this did not reach statistical significance [97]. More recent work has further suggested that increased intake of omega-3 fatty acids may exert a protective effect against RA development, particularly in individuals with a high genetic risk burden for the disease [98].

Dietary patterns may also influence RA risk. A Swedish cohort study found that higher consumption of red meat and processed meat was associated with an increased risk of RA, particularly RF-positive disease, whereas greater intake of vegetables and fruits appeared to have a protective association regardless of serological subtype [99]. In addition, Danish registry-based data indicated that heavy physical workload, especially recent heavy labor and cumulative long-term exposure, was associated with a significantly increased risk of RA in men, whereas no comparable association was observed in women, suggesting that modification of occupational exposures may represent a relevant preventive strategy in selected populations [100].

Overall, although the preventive effects of supplements and lifestyle measures are likely to be more modest than those of targeted pharmacologic interventions, their safety and feasibility make them reasonable adjunctive strategies for individuals at increased risk of RA.

## 7. Conclusions and Future Perspectives

Our understanding of preclinical RA has advanced markedly over the past 10–20 years. RA is increasingly recognized not as a disease that begins abruptly with clinically apparent synovitis, but as a continuum in which mucosal immune dysregulation, systemic autoimmunity, immune remodeling, and stromal activation evolve over time before overt arthritis becomes established. Within this framework, the management of RA is shifting from treatment of established disease toward earlier identification of at-risk individuals and therapeutic interception before irreversible joint damage occurs.

At the same time, the available evidence suggests that progression along this continuum is neither linear nor uniform. Not all individuals with autoantibodies, arthralgia, or subclinical inflammation will develop RA, and the biological processes that govern transition from reversible immune dysregulation to persistent, tissue-embedded disease remain incompletely understood. A central challenge for the field is therefore to define where along this continuum reversible immune dysregulation gives way to self-sustaining, tissue-embedded disease, because this threshold is likely to determine both the feasibility and the intensity of preventive intervention.

Accordingly, the next major goal will be to establish risk-adapted intervention strategies that align treatment intensity with the magnitude and nature of progression risk. Individuals with low-risk profiles may be best managed with surveillance and modification of environmental exposures, whereas those with convergent high-risk features—such as CSA, high-titer autoantibodies, imaging-detected tenosynovitis or synovitis, and molecular evidence of immune activation—may represent the population most likely to benefit from pharmacologic intervention. Future progress in this field will depend on refining predictive biomarkers, integrating clinical and biological risk stratification, and defining the optimal timing and mechanism of intervention. Through such efforts, prevention of RA may move from a conceptual aspiration toward a clinically achievable strategy.

## Figures and Tables

**Table 1:** Conceptual framework for risk-adapted intervention in preclinical rheumatoid arthritis.

Risk Category	Typical Features	Suggested Management Concept
Low risk	nonspecific musculoskeletal symptoms and/or low-level serological abnormalities without imaging evidence of inflammation	surveillance, lifestyle modification
Intermediate risk	CSA or limited seropositivity without clear high-risk imaging or molecular features	close monitoring, repeat imaging/biomarkers
High risk	high-titer ACPA/RF, MRI-detected tenosynovitis/synovitis, multiple convergent biomarkers	consideration of pharmacologic intervention in trial settings

* Abbreviations: ACPA, anti-citrullinated protein antibodies; CSA, clinically suspect arthralgia; RF, rheumatoid factor; MRI, magnetic resonance imaging.

**Table 2:** Summary of major interventional trials in preclinical rheumatoid arthritis.

Category	Methotrexate (MTX)	Abatacept (ABT)	Abatacept (ABT)	Abatacept (ABT)	Rituximab (RTX)	Hydroxychloroquine (HCQ)
Target population	CSA and individuals at high risk of RA onset	ACPA- and/or RF-positive individuals with inflammatory arthralgia	ACPA-positive individuals with MRI-detected inflammation	High-risk individuals	ACPA- and RF-positive individuals with arthralgia	Anti-CCP-positive asymptomatic individuals
Trial (year)	TREAT EARLIER (2022 & 2024)	APIPPRA (2024)	ARIAA (2024)	ALTO (2026)	PRAIRI (2019 & 2024)	STOP RA (2025)
Intervention	1 year of treatment	Weekly subcutaneous administration for 1 year	6 months	1 year	Single dose of 1000 mg	200–400 mg/day for 12 months
Main outcomes	Development of clinical RA; MRI findings; pain; physical function	RA incidence; arthritis-free survival	MRI findings; RA development	Time to RA onset	RA incidence; time to RA onset	RA incidence; time to RA onset
Key findings	No reduction in progression to RA; improvement in MRI inflammation and symptoms; possible reduction in RA development in ACPA-negative individuals	Significant reduction in RA incidence, with sustained benefit at 24 months	Sustained improvement in MRI inflammation and reduced risk of RA development	Delayed onset of RA by up to 4 years	No significant difference in cumulative incidence; time to onset prolonged by approximately 12 months	No significant difference between groups

* Abbreviations: CSA, clinically suspect arthralgia; RA, rheumatoid arthritis; RF, rheumatoid factor; ACPA, anti-citrullinated protein antibodies; anti-CCP, anti-cyclic citrullinated peptide antibodies; MRI, magnetic resonance imaging; MTX, methotrexate; ABT, abatacept; RTX, rituximab; HCQ, hydroxychloroquine.

## Data Availability

No new data were created or analyzed in this study.
